# The VWFA: it's not just for words anymore

**DOI:** 10.3389/fnhum.2014.00088

**Published:** 2014-03-20

**Authors:** Alecia C. Vogel, Steven E. Petersen, Bradley L. Schlaggar

**Affiliations:** ^1^Department of Psychiatry, Washington University in St. LouisSt. Louis, MO, USA; ^2^Department of Neurology, Washington University in St. LouisSt. Louis, MO, USA; ^3^Department of Radiology, Washington University in St. LouisSt. Louis, MO, USA; ^4^Department of Anatomy and Neurobiology, Washington University in St. LouisSt. Louis, MO, USA; ^5^Department of Psychology, Washington University in St. LouisSt. Louis, MO, USA; ^6^Department of Pediatrics, Washington University in St. LouisSt. Louis, MO, USA

**Keywords:** visual word form area, occipito-temporal cortex, fMRI, resting-state fMRI, resting-state functional connectivity, resting-state networks, reading, orthography

## Abstract

Reading is an important but phylogenetically new skill. While neuroimaging studies have identified brain regions used in reading, it is unclear to what extent these regions become specialized for use predominantly in reading vs. other tasks. Over the past several years, our group has published three studies addressing this question, particularly focusing on whether the putative visual word form area (VWFA) is used predominantly in reading, or whether it is used more generally in a number of tasks. Our three studies utilize a range of neuroimaging techniques, including task based fMRI experiments, a seed based resting state functional connectivity (RSFC) experiment, and a network based RSFC experiment. Overall, our studies indicate that the VWFA is not used specifically or even predominantly for reading. Rather the VWFA is a general use region that has processing properties making it particularly useful for reading, though it continues to be used in any task that requires its general processing properties. Our network based RSFC analysis extends this finding to other regions typically thought to be used predominantly for reading. Here, we review these findings and describe how the three studies complement each other. Then, we argue that conceptualizing the VWFA as a brain region with specific processing characteristics rather than a brain region devoted to a specific stimulus class, allows us to better explain the activity seen in this region during a variety of tasks. Having this type of conceptualization not only provides a better understanding of the VWFA but also provides a framework for understanding other brain regions, as it affords an explanation of function that is in keeping with the long history of studying the brain in terms of the type of information processing performed (Posner, 1978).

Reading is central to most of our lives—after all, you are reading this manuscript. Certainly reading is often an integral part of modern life, necessary for reading scientific papers, novels and news, but also important for such quotidian tasks as reading street signs, instruction sheets, prescription information, and recipes. Reading is integral to academic success (Stanovich, 1986). Clearly, reading is important, and given that the key difference between language and reading is the use of written characters, it stands to reason that reading must rely on the development of parts of the brain devoted to processing written words.

However, while reading is important, it is a relatively new, and far from universal, human skill. Written language was developed only about 5000 years ago and the printing press was invented in the mid fifteenth century. Much of the world lacks even basic literacy. In the United States up to 17% of the population are not fluent readers with reading skill at or below the 4th grade level (Stanovich, 1986; Baer et al., 2009). This functional illiteracy characterizes up to 44% of those living in poverty (Baer et al., 2009). Thus, it is extraordinarily unlikely that the capacity to read is intrinsic to the human brain or that natural selection had an opportunity to specialize brain regions for reading. The amount and kind of overt teaching and practice needed to achieve fluent reading underscores the lack of intrinsic capacity to read afforded by the human brain (Schlaggar and McCandliss, 2007).

Knowing that reading is important, yet new and not universal, leaves us with the supposition that as we develop into fluent readers we are likely to repurpose parts of the brain originally devoted to something other than reading, *per se*, and use those regions to process written characters, turn those visual representations into sounds, and extract their meanings. Yet while this supposition is widely held, there remains disagreement about the extent to which learning to read changes the brain. Does reading truly remodel the brain and result in brain regions specifically or predominantly devoted to reading (i.e., Dehaene and Cohen, 2007) or does learning to read depend on utilizing brain regions that continue to maintain other functions or processing features (i.e., Price and Devlin, 2003)?

Prior work can be found to support both of the aforementioned hypotheses, that regions of the brain become used relatively specifically for reading and that regions of the brain used in reading also continue to be used more broadly. Proponents of the former hypothesis have termed a region of the brain in left occipito-temporal (OT) cortex the visual word form area (VWFA) (McCandliss et al., 2003; Cohen and Dehaene, 2004). The argument that the VWFA is predominantly or even specifically used for words is based on both classic work demonstrating lesions of left OT cortex near the putative VWFA disrupt fluent reading (Dejerine, 1892; Cohen et al., 2003; Gaillard et al., 2006) and more recent functional magnetic resonance imaging (fMRI) experiments demonstrating the VWFA often shows more activity for words than similar non-word stimuli such as consonant strings (Cohen et al., 2002; Polk et al., 2002; Cohen and Dehaene, 2004; Baker et al., 2007; Vinckier et al., 2007). Additionally, activity in this region is not based on simple visual stimulation, as there is similar activity, as measured by fMRI, regardless of word size or font (Cohen et al., 2003; McCandliss et al., 2003). However, proponents of the second hypothesis, that the regions of the brain used in reading continue to be utilized in other types of information processing, argue that the VWFA, while used in processing words, also shows activity when processing other visual stimuli, including numbers, line drawn pictures, colors, and gratings (Tagamets et al., 2000; Price and Devlin, 2003; Xue et al., 2006; Ploran et al., 2007; Van Doren et al., 2010; Kherif et al., 2011). Several recent reviews address the body of data around this question (Dehaene and Cohen, 2011; Price and Devlin, 2011).

Recently, our group has published three studies utilizing various neuroimaging techniques, including task based fMRI experiments, a seed based resting state functional connectivity (RSFC) experiment, and a network based RSFC experiment, in an attempt to address the competing hypotheses that regions of the brain, specifically the VWFA, are used predominantly or even specifically in reading vs. the hypothesis that regions of the brain, including the VWFA, are used more generally in a number of different tasks. First, we describe a task-based fMRI experiment indicating stronger activations for non-word visual stimuli than words in left OT cortex in the same location as the VWFA. This experiment also demonstrates activity in left OT cortex is driven by other visual properties such as visual complexity and a property we call “groupability,” rather than word-likeness *per se* (Vogel et al., 2012b). Second, we describe a seed-map based RSFC experiment establishing that the VWFA has stronger resting state correlations with regions of the dorsal attention network than regions thought to be used predominantly in reading (Vogel et al., 2012a). Last, we describe network based RSFC analyses demonstrating regions thought to be used predominantly in reading have no special RSFC relationship to one another (Vogel et al., 2013). Rather than address the whole of the literature related to VWFA, we will review each of our studies in more detail below and then discuss how they relate to the larger body of work addressing the question of VWFA specificity.

## Summary of studies

### The left occipital-temporal cortex does not show preferential activity for words

As discussed above, there has been much debate about how specifically a region of left occipital-temporal cortex called the VWFA is activated by words. The region was named the visual word form area in part due to the opinion that it responded predominantly to words (McCandliss et al., 2003). However, that specificity has been debated essentially since the name VWFA was coined (Price and Devlin, 2003). We have recently published a study that compared fMRI activity elicited by a visual matching task using words, pseudowords that contained only letter combinations typically present in English words, non-words that contained letter combinations that are illegal in English, consonant strings, Amharic character strings which comprise the writing system used in Ethopia, and line drawn pictures (Vogel et al., 2012b).

**Figure 1:**
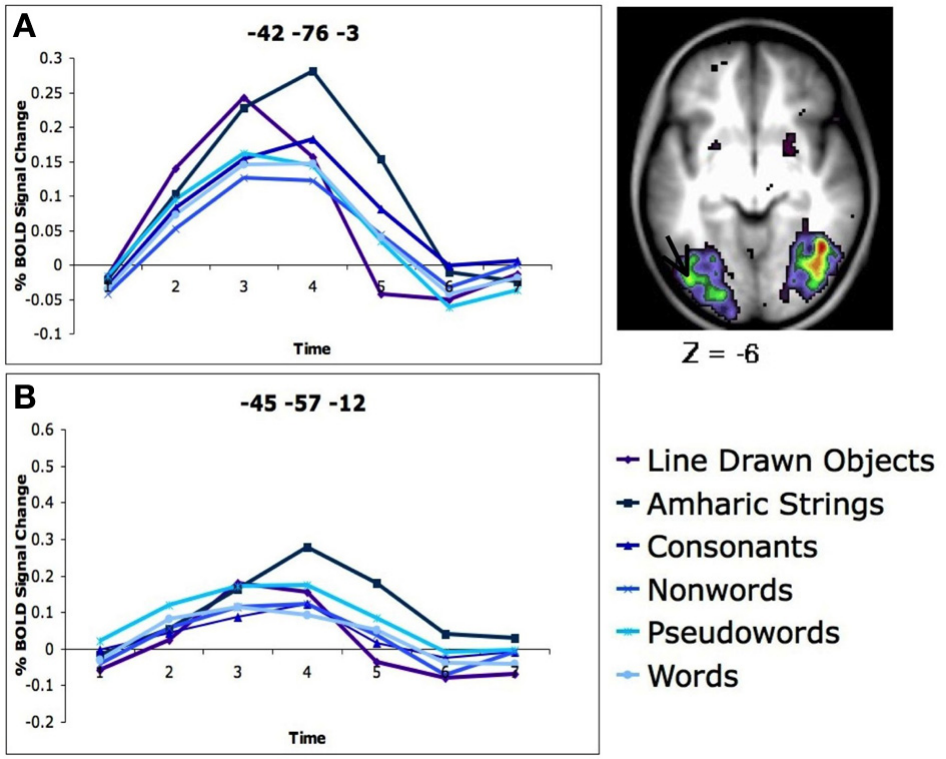
**There is more activity for Amharic character strings than letter strings in the left OT cortex**. **(A)** Activity profile and location of region of the left OT cortex defined in a whole brain analysis of stimulus type. The location of the region closest to the VWFA is denoted with an arrow. The timecourses of BOLD activity for each stimulus type is shown for this region. **(B)** Timecourses of BOLD activity for each stimulus type in an region applied to the classic VWFA coordinates (coordinates in MNI, original Talaraich coordinates taken from Cohen and Dehaene, 2004). Figure adapted from Vogel et al. (2012b).

Our study demonstrated no specificity in VWFA activity. Healthy, neurotypical, skilled adult readers were asked to determine if two simultaneously presented strings of letters, Amharic characters (described above), or line drawn pictures were the same or different and give a button press response. In a whole brain analysis looking for regions in which there was differential activity for the five types of stimuli, a region was found in left occipital-temporal (OT) cortex near the VWFA. However, in this region the activity was greatest for matching Amharic characters and line drawn pictures, which were significantly stronger than matching consonant strings, which was significantly stronger than matching non-words, pseudowords, and words (Figure 1A). When a region was applied directly on the reported coordinates of the VWFA (Cohen and Dehaene, 2004), the same pattern emerged, with the strongest activity seen for the Amharic character strings, less for consonants, line drawn pictures, non-word, pseudoword, and word stimuli (Figure 1B). Clearly, this set of results is inconsistent with the supposition that the VWFA is specifically or even predominantly used in processing words, a conception that would predict the VWFA to have the strongest activity for processing words, with less activity for the least word-like stimuli, such as Amharic characters.

Given that left OT cortex, including the VWFA, was not activated specifically or predominantly by words, in the same manuscript described above, we performed a second set of analyses designed to determine what properties do drive VWFA activity (Vogel et al., 2012b). Specifically, we hypothesized that stimuli most likely to drive left OT cortex were high spatial frequency, high contrast, complex stimuli that can be processed in groups. We chose these characteristics as they comprise some of the most salient properties of letters, words and other stimuli that have been shown to activate left OT. For example, all words, as well as numbers, line drawings, and gratings, which have been shown to activate the VWFA, are high spatial frequency and high contrast. Additionally, recent studies have shown left OT cortex to be directly responsive to spatial frequency (Kveraga et al., 2007). Unfortunately, all of our stimuli were also high spatial frequency and high contrast, so we were unable to evaluate these properties. There is also evidence that the VWFA is responsive to complexity, as patients with VWFA lesions not only have difficulty reading fluently, but also have difficulty processing more visually complex stimuli (Behrmann et al., 1998).

We were able to evaluate the effect of visual complexity on VWFA activity as our stimuli did vary in complexity, which we measured as the number of brushstrokes per character, a method previously used to compare writing systems (Changizi and Shimojo, 2005). First, we divided each string type (Amharic strings, consonant strings, non-words, pseudowords, and words) into three groups based on visual complexity, or the number of brushstrokes per character. Then, we looked for regions of the cerebrum that showed differential activity between the most complex and least complex groups. A region in left OT was found to have activity differences related to complexity, and a region based analysis demonstrated that difference was driven by increased activity for the most complex strings relative to the least complex strings in left OT.

Additionally, we were able to use reaction time data on the matching task and within stimuli properties to validate that some stimuli, like words, were processed in groups of letters, while others, such as the Amharic strings, were processed as individual characters. This “grouped” processing was also reflected in fMRI activity. It is intuitive that we read words as groups of letters; reading words as a whole or in sets of graphemes is one of the hallmarks of fluent reading (Weekes, 1997; Cohen et al., 2003). Lesions to left OT cortex are shown to result in “letter by letter” reading in which each letter of a word must be processed individually and response time increases linearly with length, accordingly (Cohen et al., 2003). However, intuition also indicates that processing unfamiliar, complex strings, such as readers who are naïve to the Amharic alphabet processing Amharic strings, requires evaluating each character of the string individually.

**Figure 2:**
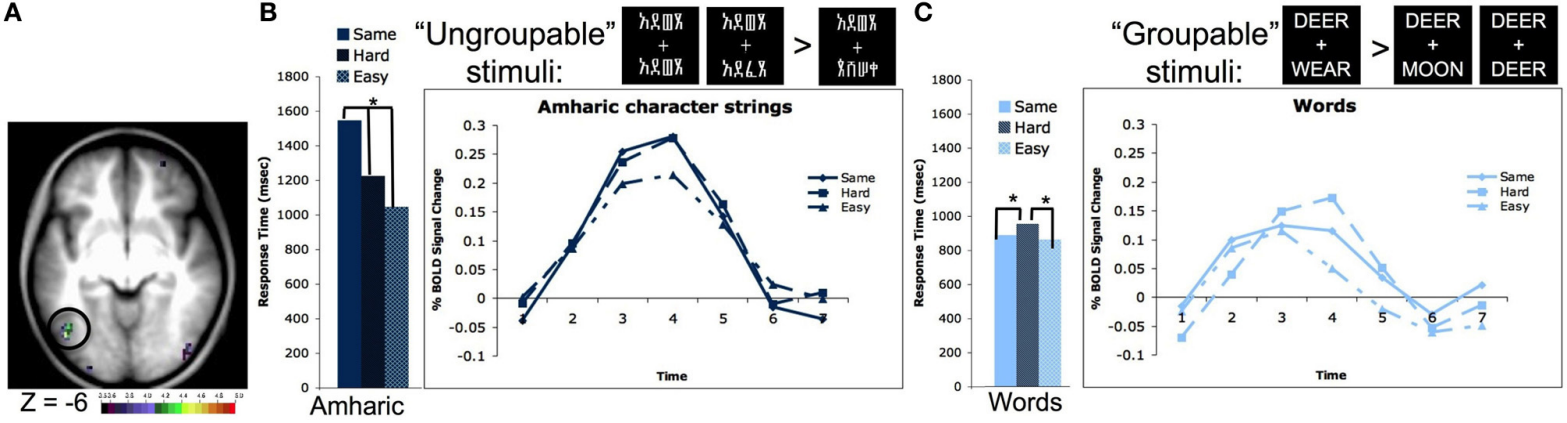
**The left OT processes unfamiliar stimulus strings as individual characters and familiar strings as groups of characters**. **(A)** Location of the left OT region defined in a whole brain pair type by timecourse analysis (−44, −67, −4 in MNI coordinates). **(B)** Reaction times and timecourses of BOLD activity for Amharic character pairs that are all the same, 2 character different hard pairs, and 4 character different easy pairs. The RTs and BOLD activity increase with the number of characters that must be evaluated to make a matching decision, indicating character by character processing. Asterisks denote RT values that have differences with *p* < 0.05. Though not shown, consonant strings show a similar pattern of both RTs and BOLD activity. **(C)** Reaction times and timecourses of BOLD activity for word pairs that are all the same, 2 character different hard pairs, and 3 character different easy pairs. The RTs and BOLD activity are equivalent for the all same and all different easy pairs, indicating these stimuli are evaluated as a group. Asterisks denote RT values that have differences with *p* < 0.05. Though not shown, pseudowords, which contain all legal letter combinations, show a similar pattern of both RTs and BOLD activity. Figure adapted from Vogel et al. (2012b).

We were able to validate these intuitions due to a second property of our four-character string stimuli. The two strings presented simultaneously contained either four identical letters or characters, differed by 2 letters or characters, or had four different letters or characters. If words are processed as whole, or at least in multi-level groups, it should make no difference whether two simultaneously presented strings are all different or all the same, it should take about the same amount of time to identify the answer (same/different). However, if one had to look at the letters individually, it would take longer to identify that two strings were the same because one would have to evaluate all four letters or characters, whereas making a decision about strings that are all different requires evaluating only one character. In our experiment, the reaction time (RT) to match strings of familiar characters such as words and pseudowords matched the proposed pattern for “grouped” processing, in that it took the same amount of time to make a same/different judgment when the two strings had all of the same letters or all different letters (RTs for words shown in Figure 2C). However, stimuli that were unfamiliar to the subjects, including consonant strings and Amharic character strings showed the other proposed effect; it took subjects longer to make a same/different judgment on the identical four character strings than on the all different four character strings of unfamiliar stimuli (RTs for Amharic characters shown in Figure 2B).

A whole brain analysis searching for regions whose activity different by pair type (i.e., all same vs. all different characters) showed a region in left OT cortex located at −44, −67, −4 in MNI coordinates (shown in Figure 2A). Planned secondary analyses showed the activity in this region also varied by stimulus type (i.e., words vs. Amharic characters) and there was an interaction between the pair type and stimulus type. Further analyses showed this interaction was driven by the same pattern described above. When matching Amharic character and consonant strings, there was more activity for matching identical strings relative to strings that differed in all characters (Amharic strings shown in Figure 2B). However, when matching words and pseudowords, there was equivalent activity for matching the identical and all different pairs (words shown in Figure 2C). While this set of results mimics the response time data, these effects remain significant even when response time was used as a regressor (Vogel et al., 2012b).

While these additional analyses demonstrated that left OT is involved in processing complex stimuli in groups, both sets of analyses primarily used whole brain approaches to determine what regions of the brain showed such effects. While this approach is the least biased way to perform these analyses, it leaves open the question of whether these effects hold in the VWFA or are even localized to the same region. Applied region of interest analyses showed the same effect of complexity and groupability, at least qualitatively, in the classically defined VWFA. More persuasive, a conjunction analysis of the three effects in question—differences between stimulus type such as words, consonant strings, and Amharic strings, differences in complexity, and differences in pair type—demonstrated that all three effects are located in the same voxels in only one place in the brain, the left OT cortex. This region of left OT cortex that had increased activity for Amharic characters and consonant strings relative to words and word-like stimuli, is more active for complex stimuli, and showed “grouped” processing of words and pseudowords but character by character processing of consonant strings and Amharic characters, was located at −46, −66, −4 (MNI coordinates), very near the VWFA.

In total, this set of analyses demonstrated that the left OT cortex, including the VWFA, does not seem to be used predominantly for processing words, as it is more strongly activated for non-word stimuli. Rather, we have demonstrated that left OT cortex at or near the VWFA is used in processing visually complex stimuli in “groups.”

### The putative visual word form area is functionally connected to the dorsal attention network

Although fMRI is useful for defining when a part of the brain is activated and studying the pattern of activity across a variety of tasks can allow for a relatively board definition of a region's processing properties, fMRI is still generally limited by experimental paradigms. Recently, resting state functional connectivity (RSFC) has been shown to operate outside of those single paradigm boundaries, as RSFC correlations seem to reflect the statistical likelihood that regions of the brain are co-activated across time, including a large number of tasks. RSFC uses correlations in large, slow changes in the BOLD signal that occur even at rest. Regions that are co-activated across a number of tasks seem to have high RSFC correlations (examined in Power et al., 2011; Yeo et al., 2011). For example, there are high RSFC correlations between members of the default mode network (Greicius et al., 2003; Fox et al., 2005), dorsal and ventral attention networks (Fox et al., 2006), attention control regions such as the previously defined fronto-parietal and cingulo-opercular networks (Dosenbach et al., 2007; Seeley et al., 2007), visual regions and motor regions (Biswal et al., 1995).

We have used RSFC correlations to query with which regions the VWFA is likely most often coactivated. If the VWFA is predominantly used for reading, it should be most often coactivated with other regions thought to be used predominantly in reading, leading to RSFC between them. However, if the VWFA is used in many types of tasks, for example if it is generally used in processing high spatial frequency, high contrast, complex stimuli in groups, it may be more commonly coactivated with other regions used in such tasks, and lead to RSFC with these other regions.

**Figure 3:**
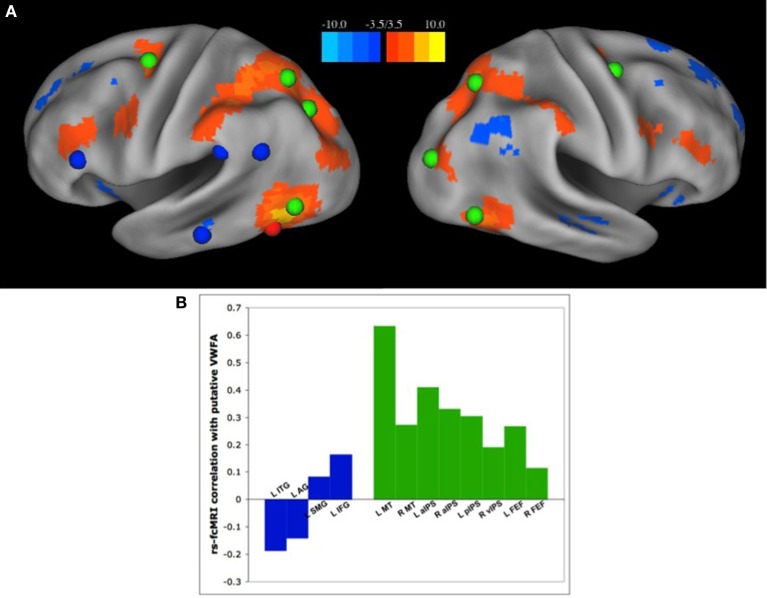
**The VWFA has stronger RSFC correlations with regions of the dorsal attention network than “reading related” regions**. **(A)** Seed map of voxels with the strongest RSFC correlations to the putative VWFA, as defined in a meta-analysis of single word reading studies. Positive correlations in RSFC are shown in warm colors, negative correlations shown in cool colors. The location of regions of the dorsal attention network are shown in green and regions thought to be used predominantly in reading, as defined by a meta-analysis of region activated by single word reading as well as a review of the literature, shown in blue. **(B)** RSFC Correlation coefficients between the VWFA and regions thought to be used predominantly in reading, shown in blue, and regions of the dorsal attention network, shown in green. Figure from Vogel et al. (2012a).

Recently, we have published a set of analyses demonstrating the latter (Vogel et al., 2012a); the VWFA has strongest RSFC correlations with regions of the dorsal attention network, and has relatively weak correlations with other regions thought to be used in reading (Figure 3). There were very weak to weakly negative correlation between the VWFA and other putatively reading-related regions, including left inferior frontal gyrus (IFG) (Fiez and Petersen, 1998; Mechelli et al., 2003) and supramarginal gyrus (SMG), thought to be used in phonological processing (Church et al., 2008, 2010), or left angular gyrus (AG) or medial temporal gyrus (MTG), thought to be related to semantic processing (Binder et al., 2005, 2009; Graves et al., 2010) (Figure 3). Additionally, the strength of RSFC correlations between the VWFA and dorsal attention regions seems to increase both with age and reading ability. In contrast, the correlations between the VWFA and putative reading regions are unrelated to age or reading level (Vogel et al., 2012a), though it should be noted that all of these analyses were performed with only movement matched groups and were not subjected to the strict quality control analyses for movement that we now know to be necessary (Power et al., 2012)

We purport that the relationship between the VWFA and regions of the dorsal attention network are related to the findings that the VWFA processes familiar stimuli, such as words, in groups. In order to process words as a whole, or in groups of letters, it is necessary to direct attention to the whole of the word or the larger group of letters. However, in order to process unfamiliar stimuli such as Amharic strings as individual characters, attention must be allocated to the individual characters. Thus, the RSFC connectivity between the VWFA and dorsal attention regions, that increases with age and reading level, reflects its more general use in processing various visual stimuli in appropriately sized groups (Vogel et al., 2012a).

### Functional network of reading-related regions across development

In addition to defining the predominant functional connections of a given region, RSFC can be used to define the network structure of large groups of regions. For example, in the analysis described above, we used the RSFC correlations between the VWFA and the rest of the brain to demonstrate that the VWFA was more commonly co-activated with regions in the dorsal attention network than other reading related regions. Alternatively, one could look at regions across the brain and attempt to discern whether there is a “reading community,” or a group of regions with strong RSFC correlations that seem to be most commonly activated in reading tasks and correspondingly, whether the VWFA is part of such a “reading community.”

Defining groups or communities of highly related items within a larger group or network of items based on a similarity metric is the purview of a branch of mathematics termed graph theory. In graph theory, graphs are defined as a group of items, also termed nodes, and the relationships between them, also called edges (Sporns et al., 2004), and this theory provides a powerful new way to define communities of brain regions using similarity as defined by RSFC correlations (Power et al., 2011). Our lab has used graph theoretic techniques on RSFC correlations to define the network structure of many general use regions across the brain and to define the network structure of the brain at the level of the MRI voxel (Power et al., 2011). These whole brain analyses have defined a number of communities previously demonstrated to be commonly co-activated across tasks, including primary visual regions, default mode regions, dorsal and ventral attention regions, cognitive control regions, as well as several previously unidentified communities (Power et al., 2011). No community of regions that could be construed as “reading related” was found in these large analyses.

While no “reading community” was defined in whole brain analyses, we wished to be certain this result was not due to either missing regions used in reading, or overwhelming any reading related effect with a whole brain analysis. Thus we performed similar graph analyses on regions defined by a meta-analysis of studies in which subjects read single words aloud (Vogel et al., 2013). Similar to the hypotheses stated above, if regions such as the VWFA are used specifically or even predominantly in reading, they should have high RSFC correlations with each other and lower RSFC correlations with other brain regions, allowing them to be grouped together into a “reading community” by the graph analytic algorithms. However, if regions used in reading are also used in a variety of other tasks, these “reading” regions will also have high RSFC correlations with the other regions with which these “reading” regions are commonly co-activated, placing them in more general communities reflecting the “reading” regions most common use (Vogel et al., 2013).

With these hypotheses in mind, we first defined all regions used in transforming a written word into spoken output via a meta-analysis of five studies of adult subjects reading single words aloud and a single developmental study of the same to capture any changes in reading activations with age (Vogel et al., 2013). The resulting group of regions included both “task general” regions, such as primary visual cortex, auditory, and motor cortex, as well as regions thought to be relatively specific to reading, including the VWFA, described above, the left SMG and IFG, thought to be related to phonological processing (i.e., Church et al., 2010), and the left AG and MTG thought to be related to semantic processing (i.e., Graves et al., 2010).

We used two graph analysis techniques, InfoMap (Rosvall and Bergstrom, 2008) and Modularity Optimization (Newman and Girvan, 2004), to define the community structure of this large network of reading-related regions across a number of RSFC thresholds, to ensure we were not biased by any one algorithm or threshold (Vogel et al., 2013). These graph analyses were performed on 3 groups of 38 subjects each, one set of adults (age 21–29 years), a set of adolescents (age 11–14 years), and a set of children (age 6–10 years) matched for image quality and movement as described in Power et al. (2012).

**Figure 4:**
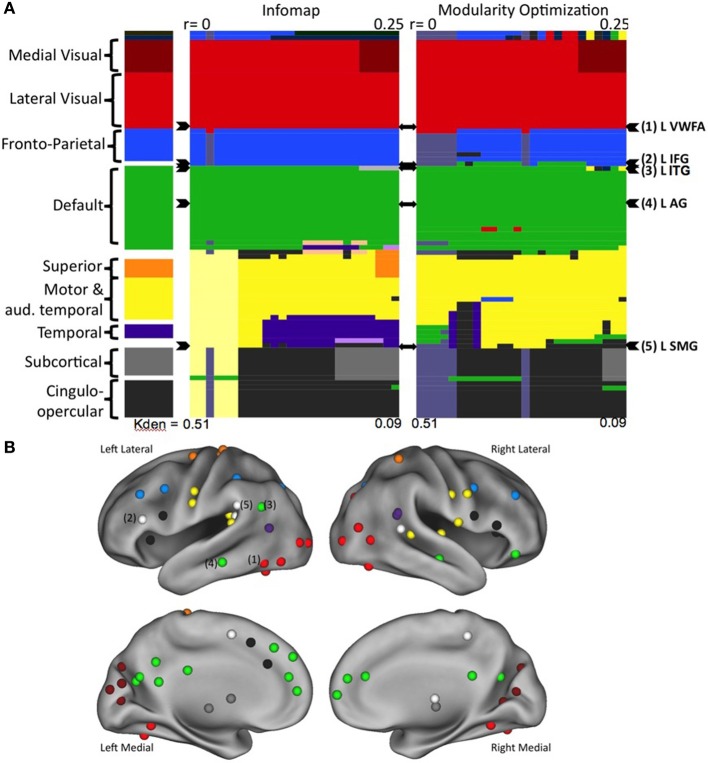
**Regions thought to be used predominantly in reading do not form a reading community in large scale network analyses**. **(A)** The network organization of all regions activated during reading. Communities were defined using two separate algorithms, the results for Infomap shown on the left and Modularity Optimization shown on the right. In each case, communities were defined across a range of RSFC correlation thresholds. Each RSFC correlation threshold corresponds to a column in the color plot. Each individual region is denoted by a row. The classification of each region is shown as the color of the row in each threshold column. The overall classification of each region, made by visual inspection of the community classification across thresholds and methods is shown in the far left column. The rows containing the regions thought of as relatively specific for reading are denoted by arrowheads to the right of the plots. **(B)** The location of all of the regions activated in a meta-analysis of reading studies, colored by overall community assignment made in the leftmost column of **(A)**. Regions thought to be relatively specifically used for reading are denoted by number. Figure from Vogel et al. (2013).

Neither InfoMap nor modularity optimization identified a “reading community” at any threshold in any age group (Vogel et al., 2013). Rather, the regions purported to be used relatively specifically for reading, including the VWFA, were largely found to be intermixed in other, more general use communities, such as visual regions, fronto-parietal and cingulo-opercular control regions, regions of default mode network (Figure 4). Additionally, there were no significant changes in the network structure of these regions-related regions across development, which included no emerging “reading community” with age/reading skill (Vogel et al., 2013). Therefore, we conclude that regions used in reading, even those thought to be essential for reading, retain more general processing properties, resulting in these regions relating to more general use communities.

## Discussion

In sum, neither functional analyses nor RSFC analyses, including both region specific and large network analyses, indicate the VWFA is used specifically or even predominantly in reading. Rather, our fMRI analyses demonstrate the VWFA is activated more strongly by non-word and even non-letter stimuli such as Amharic characters and line drawn pictures than by words, and that activity seems to be driven by other stimulus properties such as visual complexity and the “group-ability” of the stimuli (Vogel et al., 2012b). These findings are supported by the RSFC correlations of the VWFA which show stronger relationships between the VWFA and regions of the dorsal attention network than regions thought to be used predominantly in reading, likely reflecting the need to allocate spatial attention to the appropriate group of stimuli (Vogel et al., 2012a). Additionally, no reading community can be found using graph analyses to define the network structure of all regions used in reading aloud, again indicating regions used in reading retain more general processing properties (Vogel et al., 2013).

While we suggest that the VWFA has some general visual processing functions, we emphasize that we are not arguing that it is a completely general use visual region. Rather, we contend that the processing performed in the VWFA is related to specific visual properties, which can be used in processing a number of stimuli, but are also very useful for reading. For example, the VWFA is responsive to the visual complexity of stimuli, which is a shared characteristic of written languages (Changizi and Shimojo, 2005). Additionally, the VWFA processes familiar stimuli in groups, which is one of the defining features of fluent reading. In fact, lesions involving the VWFA often do not abolish reading, *per se*. Rather, they abolish fluent reading, or the ability to read words of varying lengths in about the same amount of time, while continuing to allow for “letter by letter” reading, in which words are processed as single characters (Cohen et al., 2003). We believe that this conceptualization of VWFA function, based in an information processing view of the brain, is supported not only by the results presented here, but by the wider literature, and can be used as an instructive example for understanding neural specialization more generally.

### Our results in context

The results described in this manuscript defining the VWFA as a more general use region that is particularly suited for reading due to its specific processing capabilities are largely consistent with the state of the literature. First, there has been increased acknowledgement that while the VWFA plays an important role in reading, it is not solely used for processing words. This is supported by a number of functional imaging studies (Tagamets et al., 2000; Price and Devlin, 2003; Xue et al., 2006; Ben-Shachar et al., 2007; Ploran et al., 2007; Starrfelt and Gerlach, 2007; Xue and Poldrack, 2007; Mei et al., 2010; Van Doren et al., 2010; Kherif et al., 2011), as well as lesion studies demonstrating deficits not only in reading words but also processing groups of visual stimuli or complex visual stimuli (Behrmann et al., 1990, 1998; Starrfelt et al., 2009). Moreover, we suggest that our results, that the VWFA responds to familiar stimuli in groups, may explain some of the discrepancies in the literature. As detailed in Vogel et al. (2012b), studies that demonstrate increased or specific activity for words relative to non-word stimuli typically rely on implicit or low level processing tasks (Cohen et al., 2002; Baker et al., 2007; Vinckier et al., 2007). In contrast, studies that demonstrate more activity for non-words, consonants, or symbols rely on tasks with increased processing demands (Tagamets et al., 2000; Xue et al., 2006; Xue and Poldrack, 2007; Mei et al., 2010; Van Doren et al., 2010). An important study by Brem et al. (2010) demonstrated increased activity for words in the N150 ERP response, but no corresponding increase in BOLD activity for words during an attention demanding task. All together, these results point to faster, specialized processing for words in the VWFA based on grouped processing of these familiar stimuli. However, they also demonstrate that when required to attend to non-word or even non-letter stimuli, the VWFA is also active, though likely with a slower timecourse.

While we were the first to specifically address the RSFC of the VWFA, our results can also be viewed in the context of other studies of functional connectivity, RSFC, and a recent study of structural connectivity using diffusion tensor imaging (DTI). Wang et al. (2011) described the functional relatedness of the VWFA with other parts of the brain in a visual matching task of familiar and unfamiliar stimuli. The authors demonstrated that in a visual matching task the VWFA is strongly related to the same regions of parietal cortex involved in visual attention that we see in our RSFC analysis. Additionally, Koyama et al. (2010) studied the RSFC of predefined regions thought to be involved in reading. While addressing the RSFC of the VWFA was not the foremost goal of this study, a visual inspection of the VWFA seed maps presented in the manuscript show similar results to our analysis (Vogel et al., 2012b). Finally, a recent analysis of structural connectivity using DTI demonstrated a relatively underappreciated white matter tract connecting the ventral occipital cortex near the VWFA with parietal cortex (Yeatman et al., 2013), likely in the vicinity of some of the inferior parietal lobe regions thought to be involved in visual attention.

Moreover, our results indicating the VWFA is related to other regions involved in attention processing, influencing its ability to process visual stimuli in groups, is consistent with a growing body of literature addressing the role of visual attention in fluent reading. VWFA activity in fMRI tasks was found to be related to reading skill in dyslexic children and adults in a meta-analysis by Richlan et al. (2011). Reading performance is predicted by visual attention span (Pammer et al., 2004). Furthermore, a subset of dyslexic children have a reduced visual attention span (see Valdois et al., 2004; Vidyasagar and Pammer, 2010 for reviews). These dyslexic children show deficits in simultaneous processing of consonant strings (Lassus-Sangosse et al., 2008) and meaningless non-alphanumeric strings (Lobier et al., 2012). Dyslexic adults with deficits in visual attention span also have decreased activation of both ventral occipital areas in the vicinity of the VWFA and parietal areas in multi-element alphanumeric and non-alphanumeric processing tasks (Reilhac et al., 2013). Finally, there is decreased task based connectivity between the VWFA and parietal regions in dyslexic children (van der Mark et al., 2011). Together, these results emphasize the role of the VWFA in processing visually complex stimuli of multiple types in groups, as well as emphasizing the importance of the relationship between this region and others of the dorsal attention network as detailed here.

### Processing characteristics vs. stimulus specificity

One of the major themes of the work presented here is an emphasis on defining information processing characteristics of regions rather than defining regions based on stimulus specificity. We believe this mindset is essential to understanding the brain, though we acknowledge determining how to best implement such a mindset is still up for debate. We suggest a reasoned approach is to examine past work and determine across sub-fields what kinds of stimuli or tasks are known to drive activity in a region, to look at what type of information processing, or stimulus transformations, are common across those tasks or particularly salient in those tasks. Lesion studies can be used as an adjunct to better understand what functions are disrupted when the processing done in a given injured region must be subsumed or circumvented by other parts of the brain. Finally, knowing the structural and functional connectivity of a region, what parts of the brain feed information into it, where it passes that information on to and what regions may have mediating effects on its processing, both in specific tasks and across a collective history of tasks, allow for further refinement of the types of information processing that could be carried out in a specific region. Lastly, it is useful to think of what types of processes can conceivably be carried out by a set of neurons (i.e., how could neurons reasonably represent a given stimulus or perform a given transformation or task).

This method has been very informative in our studies of the VWFA, and we argue it should be generally useful in studying the processing properties of regions across the brain. If brain regions are truly thought of as a set of neurons with given inputs and outputs, with an intrinsic organization constraining the processes performed, it becomes important to define those processing characteristics rather than limiting the consideration to the general stimulus class or task type that activates the region.

### The VWFA in only part of the ventral occipital-temporal cortex

While this review has focused on the VWFA, the VWFA is only a single region within the left OT cortex. Our group has used RSFC analyses to define the complex organization of other part parts of the brain (Cohen et al., 2008), particularly parietal cortex (Nelson et al., 2010; Barnes et al., 2012). There is increasing evidence that the organization of the left OT cortex may be even more complicated. Prior functional analyses have demonstrated that there is a gradient in activation for word-like stimuli (Vinckier et al., 2007). In addition to the visual processing described here, other studies have demonstrated effects of abstract processing or memory and semantic processing, especially in more anterior portions of the left OT, and effects of attention and cue related activity, especially in more posterior portions of the left OT (Leonards et al., 2000; Corbetta and Shulman, 2002; Egner et al., 2008; Fairhall et al., 2009). Our voxel-wise RSFC network analyses also demonstrate a complex organization of the left OT (Power et al., 2011). A number of communities are represented, including visual, dorsal attention, and fronto-parietal communities in a posterior to anterior gradient. A better understanding of this complex organization should lead to a better understanding of the processing performed in each regional component. Hopefully, a better understanding of the function of these components and their connections will also help illuminate the role or roles of the left OT in reading.

Additionally, we cannot neglect to mention that we used a group based analysis in our studies. We believe group based studies are the most reliable for studying the information processing properties of brain regions, as they allow for enough data to compare the individually defined timecourses elicited by various stimulus and task manipulations without requiring those timecourses to be fit to a model, and do not fall victim to the difficulty of correcting for multiple comparisons across each voxel of the brain. However, it is conceivable that by averaging the timecourses of multiple individuals one may “drown out” very small regions that are truly reading specific in this complicated landscape. Hopefully, as discussed above, a better understanding of the complex organization of the occipital-temporal cortex will be possible at a finer a level of detail not only for RSFC studies, but also for functional studies.

## Conclusions

In sum, our recent research on the VWFA indicates that it is not specifically or even predominantly used for reading. Rather the VWFA is a general use region that has processing properties making it particularly useful for reading, though it continues to be used in any task that requires its general processing properties. Conceptualizing the VWFA as a brain region with specific processing characteristics rather than a brain region devoted to a specific stimulus class, allows us to better explain the activity seen in this region during a variety of tasks, as well as providing an explanation of function that is in keeping with the long history of studying the brain in terms of what type of information processing is performed (Posner, 1978).

### Conflict of interest statement

The authors declare that the research was conducted in the absence of any commercial or financial relationships that could be construed as a potential conflict of interest.
